# Psychometric Properties of the HADS Measure of Anxiety and Depression Among Multiple Sclerosis Patients in Croatia

**DOI:** 10.3389/fpsyg.2021.794353

**Published:** 2021-11-30

**Authors:** Ana Jerković, Ana Proroković, Meri Matijaca, Jelena Vuko, Ana Poljičanin, Angela Mastelić, Ana Ćurković Katić, Vana Košta, Lea Kustura, Krešimir Dolić, Zoran Ðogaš, Maja Rogić Vidaković

**Affiliations:** ^1^Laboratory for Human and Experimental Neurophysiology, Department of Neuroscience, School of Medicine, University of Split, Split, Croatia; ^2^Department of Psychology, University of Zadar, Zadar, Croatia; ^3^Department of Neurology, University Hospital of Split, Split, Croatia; ^4^Institute of Physical Medicine and Rehabilitation with Rheumatology, University Hospital of Split, Split, Croatia; ^5^Department for Health Studies, University of Split, Split, Croatia; ^6^Department of Medical Chemistry and Biochemistry, University of Split School of Medicine, Split, Croatia; ^7^Department Psychiatry, University Hospital of Split, Split, Croatia; ^8^Department of Radiology, University Hospital of Split, Split, Croatia; ^9^Sleep Medical Center, University of Split, Split, Croatia

**Keywords:** Hospital Anxiety and Depression Scale (HADS), psychometrics, depression, multiple sclerosis, anxiety

## Abstract

Depression and anxiety are common complaints in patients with multiple sclerosis (MS). The study objective was to investigate the factor structure, internal consistency, and correlates of the Croatian version of the Hospital Anxiety and Depression Scale (HADS) in patients with MS. A total of 179 patients with MS and 999 controls were included in the online survey. All subjects completed the HADS and self-administered questionnaires capturing information of demographic, education level, disease-related variables, and the Multiple Sclerosis Impact Scale-29 (MSIS-29). Psychometric properties were examined by estimating the validity, reliability, and factor structure of the HADS in patients with MS. The two HADS subscales (anxiety and depression) had excellent internal consistencies (Cronbach’s α value 0.82–0.83), and factor analysis confirmed a two-factor structure. The convergent validity of the HADS subscales appeared to be good due to the significant correlations between HADS and MSIS-29. Receiver operating characteristic (ROC) analysis indicates that the HADS subscales have a significant diagnostic validity for group differentiation. Hierarchical regression analysis using MSIS-29 subscales as criterion variables showed consistent evidence for the incremental validity of the HADS. The HADS is a reliable and valid self-assessment scale in patients with MS and is suggested to be used in clinical monitoring of the psychiatric and psychological status of patients with MS.

## Introduction

Multiple sclerosis (MS) has a high prevalence of depression, anxiety, and stress comorbidities (Marrie et al., 2018; Karimi et al., 2020). Comorbid depression and anxiety disorders affect more than 20% of the MS population (Beiske et al., 2008; Fiest et al., 2015; Marrie et al., 2015, 2018; Karimi et al., 2020). Various screening instruments have been used to evaluate depression, anxiety, and stress in a clinical population of people with MS (pwMS) and non-clinical populations, including the Beck Depression Inventory-II (BDI-II) (Beck and Steer, 1990; Watson et al., 2014), Hospital Anxiety and Depression Scale (HADS) (Zigmond and Snaith, 1983; Honarmand and Feinstein, 2009), and Depression, Anxiety, and Stress Scale-21 (DASS-21) (Lovibond P.F. and Lovibond S.H., 1995; Lovibond S.H. and Lovibond P.F., 1995; Rogić Vidaković et al., 2021). The HADS is one of the most commonly used scales for assessing anxiety and depression among patients in a general hospital setting (Zigmond and Snaith, 1983; Mitchell et al., 2010). Watson et al. (2014) validated anxiety and depression measures in pwMS, confirming HADS as an appropriate questionnaire to assess depression and anxiety in pwMS. Recently Rogić Vidaković et al. (2021) reported psychometric properties of the DASS-21 scale in pwMS. The normative data for the HADS in pwMS were provided in pwMS in different languages (Honarmand and Feinstein, 2009; Atkins et al., 2012; Watson et al., 2014; Marrie et al., 2018; Pais-Ribeiro et al., 2018). A systematic review of the structure of the HADS (Cosco et al., 2012) found inconsistencies in the latent structure of the scale, which were mainly related to the different latent variable analysis methods [exploratory factor analysis and confirmatory factor analysis (CFA)] used for HADS. Regarding factor structure of HADS in pwMS MS, Pais-Ribeiro et al. (2018) conducted CFA and exploratory factor analysis providing support for a two-factor HADS structure in pwMS. There have also been specific problems in the translated versions and cross-cultural use of the HADS (i.e., authors from the same country do not apply the identical versions of HADS translations) (Wichowicz and Wieczorek, 2011; Maters et al., 2013; Watrowski and Rohde, 2014). The HADS has been validated in a diverse group of subjects, including those in primary care patients (el-Rufaie and Absood, 1995), geriatric patients (Flint and Rifat, 1996), or cancer patients (Mitchell et al., 2010). In addition, specific HADS cut-off points have been established for patients with cancer (Ibbotson et al., 1994), gynecological disorders (Abiodun, 1994), stroke (Johnson et al., 1995), and for pwMS (Honarmand and Feinstein, 2009). In previous studies conducted in Croatia, the HADS has been used in medical conditions other than MS (Filipovic-Grcic et al., 2010; Vuletić et al., 2011; Ostojić et al., 2014; Pokrajac-Bulian et al., 2015; Miljanović et al., 2017), but no study determined psychometric properties for the Croatian version of the HADS in pwMS. Two studies conducted in Croatia with HADS stated the origin of the Croatian version of HADS (Miljanović et al., 2017; Galić et al., 2020), and so far, only Miljanović et al. (2017) investigated metric properties of HADS in terminal cancer patients but having relatively smaller convenient sample size without a control group.

## Objective

The purpose of this online survey, was to evaluate the metric properties of the Croatian version of the HADS in terms of validity, reliability, and factor structure in pwMS. The study compared HADS subscales with a non-clinical population (control healthy subjects) and published data in pwMS (Watson et al., 2014; Pais-Ribeiro et al., 2018). The study also investigated the incremental validity of HADS using the Multiple Sclerosis Impact Scale-29 (MSIS-29) (Hobart et al., 2001) and relevant demographic and disease-related variables as the criterion variables.

## Materials and Methods

### Study Population and Procedure

The subjects with MS were recruited by advertising through the Association of Multiple Sclerosis Societies of Croatia (AMSSC). A total of 179 pwMS and 999 control subjects were included in the online survey. The demographic factors, education level, and disease-related factors for pwMS and control subjects are presented in Table 1. In the group of pwMS, 84% were women with a mean age of 41.3 ± 11.5 years, and 16% were men with a mean age of 42.7 ± 9.9 years. Most pwMS were right-handed (92.7%) and 35–49 years old (49%). Most pwMS had high school degrees (49.1%) and graduate university degrees (23.5%). Most of the pwMS were diagnosed with MS disease between 0 and 5 years (41.4%), 26.7% were diagnosed between 6 and 11 years, and 31.8% reported having MS over 11 years. The mean duration of the disease for pwMS was 8.7 ± 7.2. A majority of the people declared to have relapsing-remitting MS (RRMS) (70.4%), while others reported having secondary progressive MS (SPMS) (7.8%) and primary progressive MS (PPMS) (10.6%). Some pwMS (11.2%) did not provide information on the type of MS. The median Expanded Disability Status Scale (EDSS) score for all pwMS was 3.5 ± 3.5. Of the 179 pwMS, 51.8% had comorbidities, of which the most common were endocrine, nutritional, and metabolic diseases (9.9%) and diseases of the circulatory system (7.8%).

**TABLE 1:** Characteristics of study participants.

	**Control subjects (*N* = 999)**	**pwMS (*N* = 179)**
Age in years (mean ± SD)	39.9 ± 10.2	41.6 ± 11.3
Age (range)	20–74	19–75
**Sex**		
Female	81%	84%
Male	19%	16%
**MS type**		
RRMS		70.4%
SPMS		7.8%
PPMS		10.6%
Not known		11.2%
Years of MS disease (mean ± SD)		8.7 ± 7.2
EDSS (median ± IQR, range)		3.5 ± 3.5, 0–9
EDSS*		2.5 ± 2.5
EDSS**		6 ± 2
**Self-report scales (mean ± SD)**		
HADS-A	6.5 ± 3.6	8.8 ± 4.1
HADS-D	5.1 ± 3.1	7.8 ± 3.9
MSIS-29 PHYS		46.6 ± 17.2
MSIS-29 PSY		24.3 ± 8.8

SD, standard deviation; IQR, interquartile range; EDSS, Expanded Disability Status Scale; EDSS*, fully preserved mobility 0–4.5; EDSS**, partially or fully impaired mobility 5–9.5; RRMS, relapsing-remitting multiple sclerosis; SPMS, secondary progressive multiple sclerosis; PPMS, primary progressive multiple sclerosis; HADS-A, HADS Anxiety subscale; HADS-D, HADS Depression subscale; MSIS-29 PHYS, MSIS-29 Physical subscale; MSIS-29 PSY, MSIS-29 Psychological subscale.

In the group of control subjects, 81% of participants were women with a mean age of 39.8 ± 10.3 years, and 19 percent (19%) were men with a mean age of 40.3 ± 10.1 years. Most of the controls were right-handed (93.4%) and between 35 and 49 years old (51%), and most of them had graduate university degrees (43.7%) and high school degrees (25.6%). Of a total, 27.6% of people had comorbidities, of which the most common were endocrine, nutritional, and metabolic diseases (8.2%) and diseases of the circulatory system (5.2%).

The data were collected *via* a Google Forms survey from December 16, 2020, until January 13, 2021.

### Measurements and Data Collection

#### Demographic Information and Disease-Related Variables

The participants were characterized by demographic information (age, sex, and handedness), educational, and disease-related factors, including duration of the disease, MS type (Lublin et al., 2014), and the score on the EDSS (McDonald et al., 2001).

#### Hospital Anxiety and Depression Scale (HADS)

The HADS (Zigmond and Snaith, 1983) is a self-report scale consisting of two subscales, one measuring anxiety with seven items (HADS-A) and one measuring depression with seven items (HADS-D). The subject gives answers to each question on a 4-point (0–3) Likert scale and answering how he/she has been feeling in the past week. Items 1, 3, 5, 7, 9, 11, 13 belong to the anxiety subscale, while items: 2, 4, 6, 8, 10, 12, 14 belong to the depression subscale. The total score is obtained by summing the scores within each subscale. According to Pais-Ribeiro et al. (2018) interpretation, the score 0–7 represents “normal,” 8–10 “mild,” 11–14 “moderate,” and 15–21 “severe.” In the present study, the cut-off score of ≥8 and of ≥11 was used for HADS subscales (Botega et al., 1995; Bjelland et al., 2002; Honarmand and Feinstein, 2009; Brennan et al., 2010; Watson et al., 2014; Litster et al., 2016).

#### Multiple Sclerosis Impact Scale-29

The MSIS-29 is a self-report scale capturing MS disease’s impact from a patient’s physical and psychological perspective (Hobart et al., 2001; Rogić Vidaković et al., 2021). The MSIS-29 is a self-report scale capturing MS disease’s impact from a patient’s physical and psychological perspective. The scale is structured into two subscales, a 20-item scale for measuring physical impact and a 9-item scale for measuring the psychological impact of the disease. The “physical impact” subscale consists of items from 1 to 20. The subscale of “psychological impact” consists of items from 21 to 29. The patient is instructed to read each statement about the disease’s impact on his/her everyday life in the past 2 weeks. For each statement, the patient’s task is to circle the number that best describes his/her condition and answering on a 5-point Likert scale (1 = not at all, 2 = a little, 3 = moderately, 4 = quite a bit, and 5 = extremely. The patient’s scores on two subscales generated by summing individual items can be transformed to a scale of 0–100, with higher scores indicating a more severe disease burden.

#### Translation and Cultural Adaptation

Croatian translation of the HADS questionnaire was used in the evaluation of anxiety and depression in patients suffering from oncological (Miljanović et al., 2017) and neurological (Vuletić et al., 2011; Ostojić et al., 2014) diseases or other conditions (Filipovic-Grcic et al., 2010). Recently, HADS was used in the general Croatian population during the COVID-19 infection (Galić et al., 2020). Among the mentioned studies that used the Croatian translation of HADS, two studies stated the origin of the translated version of the HADS questionnaire. Miljanović et al. (2017) used the purchased Croatian translation of HADS from Mapi Research Trust, and Galić et al. (2020) used the translated Croatian version of HADS from Pokrajac-Bulian et al. (2015).

The reason why our group initiated the HADS translation procedure is the fact that the translation of HADS from Mapi Research Trust is not entirely in the spirit of the Croatian language according to the authors’ opinion, and all authors of this study agreed not to use it in the present study. Also, the Croatian version of HADS from Mapi Research Trust is not publicly free of charge to the research community. Further, since Pokrajac-Bulian et al. (2015) did not detail the process of translating HADS into Croatian, having a relatively small sample size of obese people, and the main aim of the study was not the validation of HADS in the Croatian population, we did not consider it appropriate.

Therefore, our group translated HADS following current recommendations, methodological approaches, and guidelines in the process of translating, adapting, and cross-validating instruments (Sousa and Rojjanasrirat, 2011). One author of this study (MRV) and Professor of English language (Professor Dalibora Behmen – DB, from the University of Split School of Medicine), both natives in the Croatian language, translated the HADS from English to Croatian. Next, the English language professor (DB) compared both translated versions of HADS in the Croatian language and produced the final version of the questionnaires. Another independent English language professor (University of Split) who had no insight into the original English version translated the last Croatian version of the questionnaires back into the English language, completing the final adaptation of the Croatian version of HADS used in this study (Supplementary Material).

#### Validation Procedure

Internal consistency of HADS was estimated by Cronbach’s alpha coefficients and inter-item correlations. CFA was carried out to test the validity of the two-factor and one-factor models. Data were analyzed by using the generalized least square (GLS) method and the maximum likelihood (ML) estimator. Several criteria [ML Chi-square, root mean square (RMS) standardized residual, Steiger Lind RMSEA, and McDonald non-centrality index] are reported with an emphasis on the root mean square error of approximation (RMSEA), the most commonly used fit index. Convergent validity was demonstrated by the correlation between HADS and MSIS-29 subscales. Concurrent validity was assessed by comparisons between a group of pwMS and control subjects. A receiver operating characteristic (ROC) curve was used to determine the optimum cut-off score for each HADS subscales – the score that yielded the best balance between sensitivity and specificity. Furthermore, comparisons were also provided between published data on psychometric properties of the HADS (Watson et al., 2014; Pais-Ribeiro et al., 2018). Pais-Ribeiro et al. (2018) offered psychometric properties of HADS, analyzing a sample of 380 pwMS (63.9% female; mean age 40.0 ± 10.9 years; range: 16–71 years) from the outpatient Neuroimmunology Clinic at a Central Hospital in Porto, Portugal, while Watson et al. (2014) included 34 pwMS (71%) female and (29%) male; mean age 48.5 (11.1) from England.

The incremental validity of HADS was assessed by the hierarchical regression model using the MSIS-29 and relevant demographic and disease-related factors as criterion variables. Age, sex, EDSS, type of MS, duration of the disease were entered into the first step, while the scores on HADS subscales were added in the second step.

#### Statistical Analyses

Parameters of skewness and kurtosis were tested for HADS and MSIS-29 scales. Results indicated acceptable values for the parametric statistic. Mean value comparisons between our study and published studies using HADS (Watson et al., 2014; Pais-Ribeiro et al., 2018) in pwMS and differences between relevant disease-related variables were carried using *t*-tests, Chi-square test, Mann–Whitney *U* test, Kruskal–Wallis test, and variance analysis (ANOVA). The *post hoc* Bonferroni test was calculated when using multiple comparisons. Levene’s test was used to assess the equality of variances between groups. Correlation analyses were conducted using Pearson’s r coefficient and Spearman rank-order correlation (ρ). Descriptive statistics of relevant participants’ characteristics and applied scales were summarized by *N*, percentage, mean and standard deviations, median, and interquartile range. Psychometric properties were examined by estimating internal consistency, factor structure, convergent, concurrent, and incremental validity of the HADS. In all calculations, a *p*-value of <0.05 was considered statistically significant. Data analysis was performed using the software Statistica 12.

## Results

### Overview Results

The demographic characteristics, disease-related variables, and mean results on self-rating scales (HADS and MSIS-29) of pwMS and healthy subjects are shown in Table 1. No significant sex (χ^2^ = 0.05, *p* = 0.82, *p* > 0.05) and age (*t* = −4.84, *df* = 1390, *p* > 0.05) differences were found between pwMS and control subjects. The scores on HADS depression (*t* = −2.34, *df* = 177, *p* < 0.05) and MSIS-29 physical (*t* = −2.94, *df* = 177, *p* < 0.01) subscales varied significantly by MS type in pwMS. People with RRMS type (Mean_HADS–D_ = 7.4; Mean_MSIS–PHYS_ = 43.9) were less depressed and had better physical health than people with SPMS (Mean_HADS–D_ = 10.0; Mean_MSIS–PHYS_ = 55.9) and PPMS (Mean_HADS–D_ = 10.1; Mean_MSIS–PHYS_ = 56.4). For women, HADS scores on depression subscale varied significantly with MS type. The women with RRMS were less depressed [χ^2^_(_*_*df*_*_=3)_ = 8.81; *p* < 0.05] than women with SPMS and PPMS. However, the sex differences were found in pwMS in achievement on the HADS depression subscale, indicating that the male participants have a higher depression score than females with MS (*t* = −2.10, *df* = 177, *p* < 0.05). Further, in pwMS, significant differences were found between different age groups (19–34; 35–39; and 40–75 years) for HADS depression subscales (*F* = 12.34; *p* < 0.001) and MSIS-29 physical impact subscale (*F* = 12.16; *p* < 0.001). *Post hoc* results suggest an increase in depression and poorer physical health in older pwMS than younger pwMS (*p*_younger vs. older_ < 0.001; *p*_midle age vs. older_ = 0.04; *p* < 0.05; *p*_midle age vs. younger_ = 0.04; *p* < 0.05).

Further, the participants who suffer from MS for a more extended period (more than 11 years) have poorer physical health on the MSIS-29 than those who are younger and suffer from MS for a shorter period, less than 5 years (*F* = 3.29, *p* < 0.05). Furthermore, when levels of physical health and depression were compared for types of MS (1-participants with RRMS; 2-participants with other types of MS/SPMS, PPMS, MS type not provided), a significant difference was also found (*t*_depression_ = 2.34, *df* = 177, *p* < 0.05; *t*_physical_ = −2.54, *df* = 177, *p* < 0.001). Participants with RRMS had better physical health and were less depressed than people with SPMS, PPMS, and those who did not provide information on MS type.

Table 2 presents the score classification percentages of HADS anxiety and depression subscales for pwMS and control subjects. According to score classification for the HADS depression subscale, 49.8% of the pwMS exhibited a score of ≥8 compared to 20.3% of control subjects. For HADS anxiety score, 58.6% of pwMS presented a score ≥8, compared to 34.3% of control subjects. Moreover, based on the score of ≥11, for the HADS depression subscale, 27.9% of pwMS exhibited moderate or severe depression compared to 7.4% of control subjects. For the HADS anxiety subscale, 35.7% of pwMS presented a score ≥11 compared to 13% of control subjects. The prevalence of depression in pwMS seems to be higher in comparison to anxiety.

**TABLE 2:** Score classification percentages of HADS anxiety and depression subscales for pwMS and control subjects.

	**Control subjects**		**pwMS**	
	**HADS-A (%)**	**HADS-D (%)**	**HADS-A (%)**	**HADS-D (%)**
(0–7) normal	65.7	79.7	41.4	50.3
(8–10) mild	21.3	12.9	22.9	21.8
(11–14) moderate	9.6	7.2	26.8	24.0
(15–21) severe	3.4	0.2	8.9	3.9
≥8	34.3%	20.3%	58.6%	49.8%
≥11	13%	7.4%	35.7%	27.9%

*HADS-A, HADS Anxiety subscale; HADS-D, HADS Depression subscale.*

### Psychometric Properties of the Hospital Anxiety and Depression Scale (HADS)

#### Internal Consistency

Expressed by Cronbach’s ɑ coefficients, both HADS subscales (α_HADS–A_ = 0.82 to α_HADS–D_ = 0.83) and MSIS-29 subscales (α_MSIS–PHYS_ = 0.82 to α_MSIS–PSY_ = 0.81) had excellent internal consistency. Values for both HADS and MSIS-29 scales are considered indicative of good reliability. Inter-item correlations for HADS and MSIS-29 scales were >0.3, meaning that all items on each subscale correlate very well with the scale overall.

#### Factor Analysis of the Hospital Anxiety and Depression Scale (HADS)

**FIGURE 1:**
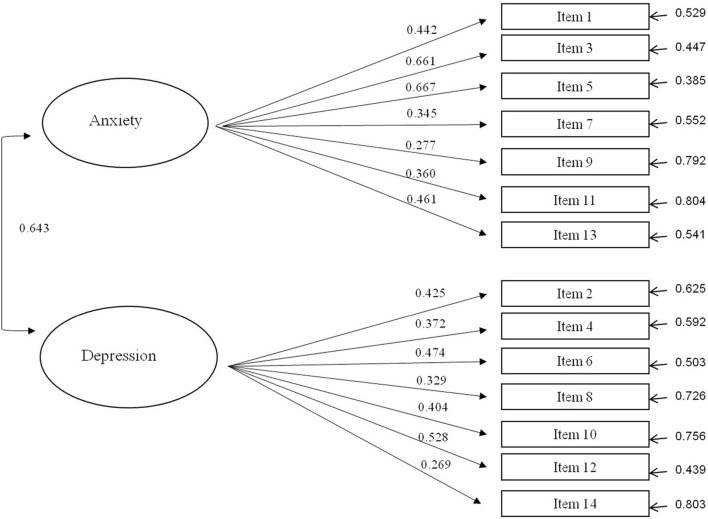
Path diagram for the confirmatory factor analysis of HADS with standardized regression weights.

Indicated by almost all obtained fitting parameters except for a slightly higher ratio between Chi-square and corresponding *df* (Kenny, 2020), CFA confirmed the original structure of the HADS in general (Figure 1 and Table 3). Namely, HADS, as expected, shows a primarily two-factor structure (separate dimensions of anxiety and depression) with mutually significantly correlated factors. HADS-A subscale explained 18.66% of factor variance and with HADS-D subscale 21.85% of the variance. The CFA for the one-factor solution was also reported (Table 3), but all fit indices support the retention of the two-factor solution. The Steiger Lind RMSEA index was used as the main and most commonly used criteria for accepting models. Cut-off RMSEA value of <0.05 indicates a “close fit,” and that <0.08 suggests a reasonable model–data fit (e.g., Browne and Cudeck, 1993; Jöreskog and Sörbom, 1993).

**TABLE 3:** Fit indices for one-factor and two-factor model of HADS (CFA).

	**One-factor solution**	**Two-factor solution**
ML Chi-square	752.03 (*df* = 77)	128.315 (*df* = 28)
RMS standardized residual	0.059	0.024
Steiger Lind RMSEA	0.107	0.051
McDonald non-centrality index	0.643	0.93

*Rms, root mean square; Rmsea, root mean square error of approximation; Ml, maximum likelihood.*

#### Convergent Validity of the Hospital Anxiety and Depression Scale (HADS)

Convergent validity was demonstrated by the correlations of the HADS subscales and the MSIS-29 subscales (Table 4) for pwMS. HADS anxiety and depression subscales have a significant moderate correlation (*r* = 0.54; *p* < 0.001). Moreover, both HADS subscales are correlated with MSIS-29 subscales, noting that the correlations of HADS subscales are higher with the psychological MSIS-29 subscale (*r* = 0.61–0.69; *p* < 0.01) compared to the physical MSIS-29 subscale (*r* = 0.33–0.54; *p* < 0.01). Correlation coefficients between HADS subscales and MSIS-29 subscales indicate weak and moderate correlations.

**TABLE 4:** Pearson correlation coefficient for HADS and MSIS-29 scale (*N* = 179).

	**HADS-A**	**HADS-D**	**MSIS-29 PHYS**	**MSIS-29 PSY**
HADS-A	–	0.54**	0.33**	0.69**
HADS-D		–	0.54**	0.61**
MSIS-29 PHYS			–	0.57**
MSIS-29 PSY				–

**p < 0.01; HADS-A, HADS Anxiety subscale; HADS-D, HADS Depression subscale; MSIS-29 PSY-MSIS-29 Psychological subscale.

#### Concurrent Validity

**FIGURE 2:**
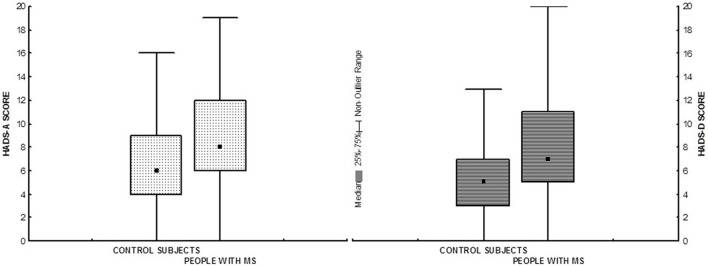
Box plots showing medians and interquartile range of HADS scores in pwMS and control subjects. HADS-A, HADS Anxiety subscale; HADS-D, HADS Depression subscale.

Concurrent validity was demonstrated by differences between MS and control subjects. HADS mean values for pwMS were significantly higher (Mann–Whitney *U* test; *z*_anxiety_ = 6.98, *p* < 0.01; *z*_depression_ = 8.588, *p* < 0.01) than those reported in control subjects (Figure 2). A non-parametric test was done because Levene’s test for homogeneity of variances was significant (both HADS-A and HADS-D). Further, compared to the results of the current study with Watson et al. (2014) and Pais-Ribeiro et al. (2018), depression and anxiety were not equally represented (Table 5). The results on both subscales were significantly higher in our sample than those presented by Pais-Ribeiro et al. (2018), and the difference is significantly more pronounced when it comes to HADS-D. Compared to Watson et al. (2014), there were no significant differences in depression levels, while the difference in anxiety exists (small effect size).

**TABLE 5:** The HADS results from the present study and comparisons between published studies.

		**HADS-A**	**HADS-D**
Present study *N* = 179	Mean (SD)	8.82 (4.11)	7.80 (3.99)
Watson et al. (2014) *N* = 34	Mean (SD)	7.2 (5.4)	8.1 (5.9)
	*t*	1.99	0.37
	*df*	211	211
	*p*	0.04; *p* < 0.05	0.71; *p* > 0.05
Pais-Ribeiro et al. (2018) *N* = 380	Mean (SD)	7.94 (4.31)	5.63 (4.01)
	*t*	2.29	5.98
	*df*	557	557
	*p*	0.02; *p* < 0.5	0.00; *p* < 0.001

*HADS-A, HADS Anxiety subscale; HADS-D, HADS Depression subscale.*

Receiver operating characteristic analysis (Table 6) indicated that for the HADS-A, the highest value of the Youden Index (*J* = 0.245) was obtained for a cut-off point of >7 and the HADS-D at the cut-off point of >6 (*J* = 0.328). For the HADS-A, the statistically significant AUC was 0.664 (*p* < 0.001) with a 95% confidence interval of 0.635–0.692. For the HADS-D, AUC was 0.702 (*p* < 0.001) with 95% confidence interval 0.675–0.728. Both parameters (*J* and AUC) indicate that the HADS-A and the HADS-D have a significant diagnostic validity for group differentiation.

**TABLE 6:** Psychometric properties of HADS-A and HADS-D at different cut-off scores (ROC analysis).

	**Sensitivity (95% CI)**	**Specificity (95% CI)**	**+LR**	**−LR**
**HADS-A scores**				
≤6	65.92 (58.5–72.8)	54.44 (51.1–57.7)	1.45	0.63
≤7	58.66 (51.1–66.0)	65.39 (62.2–68.5)	1.69	0.63
≤8	48.04 (40.5–55.6)	73.38 (70.4–76.2)	1.81	0.71
≤9	43.02 (35.7–50.6)	80.94 (78.2–83.4)	2.26	0.70
≤10	35.75 (28.7–43.2)	87.19 (84.8–89.3)	2.79	0.74
≤11	27.37 (21.0–34.5)	90.25 (88.1–92.1)	2.81	0.80
≤12	20.11 (14.5–26.7)	93.32 (91.5–94.9)	3.01	0.86
**HADS-D scores**				
≤6	61.45 (53.9–68.6)	71.37 (68.5–74.2)	2.15	0.54
≤7	49.72 (42.2–57.3)	79.58 (76.9–82.0)	2.43	0.63
≤8	43.58 (36.2–51.2)	85.39 (83.0–87.5)	2.98	0.66
≤9	35.20 (28.2–42.7)	89.19 (87.1–91.0)	3.26	0.73
≤10	27.93 (21.5–35.1)	92.49 (90.7–94.0)	3.72	0.78
≤11	21.23 (15.5–28.0)	96.30 (94.9–97.4)	5.73	0.82
≤12	12.85 (8.3–18.7)	98.70 (97.8–99.3)	9.87	0.88

*+, LR likelihood ratio for a positive result; −, LR likelihood ratio for a negative result; CI, confidence interval.*

#### Incremental Validity

Table 7 represents the results of multiple hierarchical regression analyses and the incremental validity of the HADS. Results indicate whether HADS-A and HADS-D contribute to the explanation of MSIS-29 variance (incremental validity) in relation to some examined sociodemographic variables, MS type, and EDDS.

**TABLE 7:** Multiple hierarchical regression analyses for the incremental validity of HADS and relevant variables on MSIS-29 subscales.

		**MSIS-29 PHYS**		**MSIS-29 PSY**	
		**Step 1**	**Step 2**	**Step 1**	**Step 2**
**Predictors**		**β**	**β**	**β**	**β**
Step 1	Age	0.07	0.05	−0.37**	−0.40*
	Sex	0.32**	0.22*	0.16	0.01
	Duration of the disease	0.17	0.10	0.11	0.01
	Type MS	0.11	0.14	0.01	0.04
	EDSS	0.05	0.08	0.05	0.09
Step 2	*R* ^2^	0.19		0.13	
		*F*(5,80) = 3.71 *p* < 0.001 *F*(5,80) = 2.50 *p* < 0.04			
	HADS-A		0.14		0.20*
	HADS-D		0.36**		0.54**
	*R* ^2^		0.37		0.54
		*F*(7,79) = 6.52 *p* < 0.001 *F*(7,78) = 13.00 *p* < 0.001			
	Δ*R*^2^		0.18		0.40
		*F*(7,79) = 11.18 *p* < 0.001 *F*(7,78) = 34.36 *p* < 0.001			

HADS-A, HADS Anxiety subscale; HADS-D, HADS Depression subscale; MSIS-29 PHYS, MSIS-29 Physical subscale; MSIS-29 PSY, MSIS-29 Psychological subscale; β, standardized regression coefficient; *R*^2^, coefficient of determination; Δ*R*^2^, change in the coefficient of determination; *p < 0.05, CI = 95%; **p < 0.01, CI = 98%.

For the physical impact on the MSIS-29, the first set of predictor variables (age, sex, EDSS, MS type, and disease duration) only sex had a significant β coefficient. Step 2, which included HADS subscales, revealed that these variables contribute to the explanation of an additional 18% of physical impact variance. Among these predictors, only HADS depression had significant β, which is positive, meaning that the greater depression is accompanied by greater physical impact (Table 7). For the psychological impact on the MSIS-29, age, among predictors included in the first step, significantly predicted psychological impact, accounting for 13% of the variance. Simultaneously, HADS depression and anxiety subscales entered in the second step explained 40% of the psychological impact variance. Anxiety and depression subscale significantly contributed to the explanation of the criterion variable. For both criterion variables (MSIS-29 physical and psychological impact) HADS has been shown to have significant incremental validity in the explanation of MSIS-29, especially when it comes to the second criterion, MSIS-29 psychological impact. The additional contribution of physical impact is 13%, and for psychological impact, even 40%.

## Discussion

Anxiety and depressive disorders are among the most common psychiatric illnesses highly comorbid with each other and considered to belong to the broader category of internalizing disorders (Kalin, 2020). More than 50% of the patients with major depression have significant anxiety and were considered to have anxious depression (Fava et al., 2004; Beijers et al., 2019). When looking into a healthy population compared to pwMS in terms of developing mood disorders, the risk of depression, anxiety, and stress are higher in MS patients than in healthy subjects (Pham et al., 2018). The etiology of MS disease is not yet known and factors such as immune system deficiency, genetic predisposition, lack of vitamin D, Epstein-Barr virus, family background, geographical region, stress, and lifestyle play a role in this disease (Dehghani and Kazemi Moghaddam, 2015). Besides mood disorders, relevant clinical symptoms of MS include disturbances in motor functions (e.g., tremor, weakness, and spasticity), sensory deficits (e.g., pain), visual impairments (e.g., diplopia and optic neuritis), vascular dysfunctions, obesity, and cognitive impairments (e.g., attention deficits, working memory impairments, information processing). Karimi et al. (2020) investigated 87 MS patients in Iran and showed that 47.1% had moderate depression, 39.1% had moderate anxiety, and 44.8% had moderate stress. A study in Canada (Pham et al., 2018) showed 30% of MS patients suffered from anxiety, and 16.3% were affected with depression. The results of a study in the United States (Boeschoten et al., 2017) revealed 20.6% of MS patients suffered from depression. A significant factor responsible for MS relapses is stressful life events (Brown et al., 2005; Stamoula et al., 2021). From a clinical point of view, it is therefore recommended to monitor psychological constructs such as depression, anxiety, and stress in pwMS (Glaser et al., 2019). According to a literature search, it is evident that scales such as DASS-21 (Lovibond P.F. and Lovibond S.H., 1995; Lovibond S.H. and Lovibond P.F., 1995) and HADS (Zigmond and Snaith, 1983) were mainly used for detecting depression, anxiety, and stress in pwMS. Recently psychometric properties for DASS-21 were published in pwMS (Rogić Vidaković et al., 2021), while psychometric properties for HADS in pwMS have been available on different languages from earlier years (Honarmand and Feinstein, 2009; Atkins et al., 2012; Watson et al., 2014; Marrie et al., 2018; Pais-Ribeiro et al., 2018). What it has to bear in mind is that HADS was not initially developed in pwMS. Instead, it is created as a self-report rating scale for evaluating depression and anxiety in patients with a general medical condition, but can be regarded as a useful screening instrument to detect potential psychological disturbances in pwMS (Honarmand and Feinstein, 2009; Watson et al., 2014).

By exploring the factor structure of the HADS, the present study confirmed a two-dimensionality of the HADS in a large community and patient samples (Mykletun et al., 2001; Norton et al., 2013), as well as in samples of pwMS (Pais-Ribeiro et al., 2018). Internal consistency, using Cronbach’s alpha, for the two dimensions was good, 0.80 for anxiety and 0.81 for depression in the study of Pais-Ribeiro et al. (2018), while in the present study, the Cronbach’s alpha, for the two dimensions was also good, 0.82 for anxiety and 0.83 for depression. A systematic review study conducted by Cosco et al. (2012) pointed out that previous findings on the latent structure of the HADS have been somewhat inconsistent factor structure with 25 of the 50 reviewed studies revealing a two-factor structure, 5 studies revealing unidimensional, 17 studies revealing three-factor, and 2 studies revealing four-factor structures. According to the findings of Cosco et al. (2012), different latent variable analysis methods gained correspondingly different structures: exploratory factor analysis studies revealed primarily two-factor structures, CFA studies revealed primarily three-factor structures, and item response theory studies revealed primarily unidimensional structures. Regarding factor structure of HADS in MS research, Pais-Ribeiro et al. (2018) conducted CFA and exploratory factor analysis and provided support for the bifactor model. The present study confirmed a two-factor structure, and several fit indices that were used support the retention of the two-factor solution.

Parameters of ROC analysis indicate that the HADS-A and the HADS-D have a significant diagnostic validity for group differentiation. Although the HADS depression scale shows slightly better concurrent validity than HADS anxiety, the accuracy of both measures to distinguish emotional disorder is not very high. Therefore, the present study provided data for the optimum cut-off score of >7 for HADS-A and a cut-off score of >6 for HADS-D. The cut-off score of >7 for HADS-A is similar to findings of Nicholl et al. (2001) and Honarmand and Feinstein (2009), while the cut-off score of >6 for HADS-D was slightly lower compared to other studies using HADS in pwMS (Honarmand and Feinstein, 2009; Watson et al., 2014). When looking into studies using HADS in different samples of patients (not including pwMS) like cancer patients or psychiatric illnesses, the sensitivity and specificity of HADS-A and HADS-D with a threshold of 8+ were most often found to be in the range of 0.70–0.90. The variation in optimal cut-off values and sensitivity and specificity might be due to differences in HADS translations used, samples and procedures in administration, and method analysis of HADS (Bjelland et al., 2002; Cosco et al., 2012).

Both HADS subscales had excellent internal consistencies and good convergent validity expressed by inter-correlations between the HADS and the MSIS-29 subscales. Results of regression analysis suggest that the HADS showed incremental validity in relation to age, sex, MS type, and EDSS.

Further, we have to acknowledge several limitations of the study. The possible limitation of the study would be the time of conducting the survey. Namely, the study was conducted during the COVID-19 pandemic (1 year after the first lockdown in Croatia) and a series of earthquakes that hit Croatia, causing specific problems regarding the governmental social distancing measures and collective trauma effects. Although the study was conducted during COVID-19 disease and strong earthquakes in the eastern part of Croatia (Perinja and Zagreb region), we assume that COVID-19 and earthquakes did not significantly affect the HADS results in pwMS and control subjects. Galić et al. (2020) assessed depression and anxiety in the general population with HADS 3 weeks after the first registered cases of COVID-19 in Croatia. In line with the study of Galić et al. (2020), observed values of depression were similar to the results of control subjects in the present study, with less pronounced anxiety in the present study. Further, a comparison with the previous studies shows a higher prevalence of depression and anxiety in pwMS independently of specific external factors not related to the MS disease (Dahl et al., 2004; Karimi et al., 2020). Another possible limitation is that HADS was not used as a paper–pencil assessment but rather as an online survey. The advantage of the online survey was the possibility to reach a higher number of MS patients. The paper–pencil assessment of HADS would last longer since we could access the MS patients once a week at the University Hospital of Split during the regular control examinations at the Department of Neurology. An approximate number of MS patients that we could reach weekly would be approximately three to five. The second problem was that during regular control visits at the Department of Neurology, the MS patients are not registered at specific hours but are intermingled with other patients having other neurological diseases. Therefore, we believe by conducting an online survey, we reached a satisfactory number of MS patients in a shorter period and got a more appropriate sample size avoiding possible erroneous findings which might occur in the process of determining psychometric properties of the HADS, in particular the identification of the correct structure of the questionnaire (e.g., number of dimensions and items in each dimension).

## Conclusion

The HADS is shown to be a reliable and valid patient-self report scale that captures meaningful psychological and physical clinical correlates of MS disease.

## Data Availability Statement

The original contributions presented in the study are included in the article/Supplementary Material, further inquiries can be directed to the corresponding author.

## Ethics Statement

The studies involving human participants were reviewed and approved by the School of Medicine, University of Split. The patients/participants provided their written informed consent to participate in this study.

## Author Contributions

AJ: conceptualization, data curation, formal analysis, methodology, project administration, resources, and writing – original draft. APr: supervision, methodology, and writing – original draft. MM, KD, and ZÐ: supervision and writing – original draft. JV, AM, AĆ, VK, and LK: methodology, project administration, and resources. APo: methodology and project administration. MRV: conceptualization, data curation, formal analysis, methodology, project administration, resources, supervision, and writing – original draft. All authors contributed to the article and approved the submitted version.

## Conflict of Interest

The authors declare that the research was conducted in the absence of any commercial or financial relationships that could be construed as a potential conflict of interest.

## Publisher’s Note

All claims expressed in this article are solely those of the authors and do not necessarily represent those of their affiliated organizations, or those of the publisher, the editors and the reviewers. Any product that may be evaluated in this article, or claim that may be made by its manufacturer, is not guaranteed or endorsed by the publisher.
