# Supplementary material for: Psychometric Properties of the HADS Measure of Anxiety and Depression Among Multiple Sclerosis Patients in Croatia
Source: Front Psychol. 2021 Nov 30;12:794353. doi: 10.3389/fpsyg.2021.794353 (PMC8670005; doi:10.3389/fpsyg.2021.794353)
Supplement: Supplementary file 1 [file Data_Sheet_1.docx]

Supplementary Material

**HOSPITAL ANXIETY AND DEPRESSION SCALE (HADS)**

***Translation to the Croatian language***

*Ovaj upitnik će pomoći liječniku da sazna kako se osjećate. Pročitajte svaku rečenicu. Zaokružite onaj odgovor koji najbolje opisuje kako ste se osjećali tijekom prošlog tjedna. Nemojte puno razmišljati tijekom ispunjavanja upitnika. U ovom upitniku su važniji spontani odgovori. Označite samo jedan odgovor za svako pitanje.*

| A | 1. Osjećam se napeto ili uznemireno: | 8. Osjećam se usporeno: | D |
| --- | --- | --- | --- |
| 3  2  1  0 | a. Većinu vremena  b. Često  c. Povremeno  d. Nikada | a. Gotovo cijelo vrijeme  b. Vrlo često  c. Povremeno  d. Nikada | 3  2  1  0 |
| D | 2. Još uvijek uživam u stvarima u kojima sam prije uživao/la: | 9. Obuzme me nekakav zastrašujući osjećaj poput „leptirića“ u trbuhu: | A |
| 0  1  2  3 | a. Definitivno jednako kao prije  b. Ne baš toliko kao prije  c. Puno manje nego prije  d. Gotovo nimalo | a. Nikada  b. Povremeno  c. Prilično često  d. Vrlo često | 0  1  2  3 |
| A | 3. Obuzme me nekakav zastrašujući osjećaj kao da će se nešto užasno dogoditi: | 10. Izgubio(la) sam zanimanje za svoj izgled: | D |
| 3  2  1  0 | a. Da, definitivno i prilično je jak  b. Da, ali nije prejak  c. Da, malo, ali me ne zabrinjava  d. Nikada | a. Da, definitivno  b. Ne brinem se koliko bih trebao  c. Možda se ne brinem dovoljno  d. Brinem se jednako kao i uvijek | 3  2  1  0 |
| D | 4. Mogu se nasmijati i vidjeti humor u situaciji: | 11. Osjećam se nemirno kao da moram biti u pokretu: | A |
| 0  1  2  3 | a. Jednako kao i prije  b. Nešto rjeđe  c. Dosta rjeđe  d. Nikada | a. Da, jako  b. Prilično  c. Donekle  d. Nimalo | 3  2  1  0 |
| A | 5. Zabrinjavajuće misli mi prolaze kroz glavu: | 12. S užitkom se radujem stvarima: | D |
| 3  2  1  0 | a. Većinu vremena  b. Često  c. Povremeno  d. Samo ponekad | a. Koliko i prije  b. Nešto manje nego prije  c. Definitivno manje nego prije  d. Gotovo nimalo | 0  1  2  3 |
| D | 6. Osjećam se veselo: | 13. Iznenada me uhvati osjećaj panike: | A |
| 0  1  2  3 | a. Većinu vremena  b. Često  c. Rijetko  d. Nikada | a. Jako često  b. Prilično često  c. Povremeno  d. Nikada | 3  2  1  0 |
| A | 7. Osjećam se ugodno i opušteno: | 14. Mogu uživati u dobrom TV ili radijskom programu ili knjizi: | D |
| 0  1  2  3 | a. Uvijek  b. Često  c. Rijetko  d. Nikada | a. Često  b. Ponekad  c. Rijetko  d. Gotovo nikada | 0  1  2  3 |

**Hospital Anxiety and Depression Scale (HADS)**

***Original version on the English language***

| A | 1. I feel tense or 'wound up': | 8. I feel as if I am slowed down: | D |
| --- | --- | --- | --- |
| 3  2  1  0 | a. Most of the time  b. A lot of the time  c. From time to time, occasionally  d. Not at all | a. Nearly all the time  b. Very often  c. Sometimes  d. Not at all | 3  2  1  0 |
| D | 2. I still enjoy the things I used to  enjoy: | 9. I get a sort of frightened feeling like  'butterflies' in the stomach: | A |
| 0  1  2  3 | a. Definitely as much  b. Not quite so much  c. Only a little  d. Hardly at all | a. Not at all  b. Occasionally  c. Quite Often  d. Very Often | 0  1  2  3 |
| A | 3. I get a sort of frightened feeling as if  something awful is about to  happen: | 10. I have lost interest in my appearance: | D |
| 3  2  1  0 | a. Very definitely and quite badly  b. Yes, but not too badly  c. A little, but it doesn't worry me  d. Not at all | a. Definitely  b. I don't take as much care as I should  c. I may not take quite as much care  d. I take just as much care as ever | 3  2  1  0 |
| D | 4. I can laugh and see the funny side  of things: | 11. I feel restless as I have to be on the  move: | A |
| 0  1  2  3 | a. As much as I always could  b. Not quite so much now  c. Definitely not so much now  d. Not at all | a. Very much indeed  b. Quite a lot  c. Not very much  d. Not at all | 3  2  1  0 |
| A | 5. Worrying thoughts go through my  mind: | 12. I look forward with enjoyment to  things: | D |
| 3  2  1  0 | a. A great deal of the time  b. A lot of the time  c. From time to time, but not too often  d. Only occasionally | a. As much as I ever did  b. Rather less than I used to  c. Definitely less than I used to  d. Hardly at all | 0  1  2  3 |
| D | 6. I feel cheerful: | 13. I get sudden feelings of panic: | A |
| 0  1  2  3 | a. Most of the time  b. Sometimes  c. Not often  d. Not at all | a. Very often indeed  b. Quite often  c. Not very often  d. Not at all | 3  2  1  0 |
| A | 7. I can sit at ease and feel relaxed: | 14. I can enjoy a good book or radio or TV  program: | D |
| 0  1  2  3 | a. Definitely  b. Usually  c. Not Often  d. Not at all | a. Often  b. Sometimes  c. Not often  d. Very seldom | 0  1  2  3 |

**Hospital Anxiety and Depression Scale (HADS)**

*Scoring and interpretation*

**Scoring** *(indicated in the table*):

Anxiety items: 1, 3, 5,7, 9, 11, 13

Depression items: 2, 4, 6, 8, 10, 12, 14

**Total score:**  Anxiety:________ Depression:__________

**Interpretation** *(according to Pais-Ribeiro et al. Neuropsychiatric Disease and Treatment 2018;14;3193-3197)*

| 0-7 | Normal |
| --- | --- |
| od 8 do 10 | Mild |
| od 11 do 14 | Moderate |
| od 15 do 21 | Severe |
